# Excessive Free Medical Relief

**Published:** 1907-01-05

**Authors:** T. W. S. Paterson

**Affiliations:** 42 Holland Park, London, W.


### Editor's Letter-Box.

[Our Correspondents are reminded that prolixity is a great bar to publication, and that brevity of style and conciseness of statement greatly facilitate early insertion.]

### EXCESSIVE FREE MEDICAL RELIEF.

Sir,—I read with much interest Sir Henry Burdett's speech at the Hospital Officers' Association, as reported in to-day's *Telegraph*. The thanks of the profession are due to him for the stand he is taking up.

It is most disheartening that at the time of one's life when one has the inclination and the energy to work for small fees to find that the enormous so-called charitable institutions are in direct competition with the medical man. The general practitioner now is, in many cases, thoroughly competent to treat all disease except major operations. In my experience the special hospitals are the worst offenders, especially those which charge a small fee for medicine, etc.

Some years ago I used as anæsthetist to assist a doctor who was practising on the south side of the water. The operations were mostly minor operations on the throat and nose. The fee charged was 30s. to £2 2s., of which my portion was 10s. 6d.

One day he took me to a double-fronted shop in Walworth. On my inquiring what kind of a shop it was, as I saw nothing but well-filled sacks, I was informed the proprietor was a wholesale sweet-seller; it is true he was wearing a frock-coat so green with age that he might have been regarded as a suitable patient by any hospital authority. The operation was performed on his daughter for the usual small fee. I remonstrated with my friend on the smallness of the fee, pointing out that the proprietor was probably better off than either of us would ever be from the practice of our profession. He assured me that he could not get a larger fee, as if he did not do it at her father's house he would have to do it for nothing, so far as he was concerned, at the —— Hospital, to which he was attached; in fact, she had already paid £8 to the institution for treatment, etc. This is not an isolated case. I have frequently been informed on mentioning a reasonable fee for an operation, considering the house rent they pay, etc., that if I can't do it for so much they must go to the hospital.

About the same time I saw reported in the papers a man, described as a costermonger, charged with brutally assaulting a girl who had assisted the police in his capture. He was in danger of getting six months' hard, but he was remanded to enable him to be represented. He was represented by a leading K.C.—Mr. Horace Avory, I think. I do not suppose counsel reduced his fee from the circumstances of the case. The point which surprised me was that a costermonger could find the cash to be so ably represented. We are constantly hearing of appeals from the various hospitals for funds to carry on the increased work. The London Hospital is not behindhand in this respect. In these days of statistics it would be interesting if the authorities of the London Hospital would publish the number of in-patients in 1905 and the number of patients received as in-patients during that period, who resided outside a radius of the London Hospital of, say, 20 miles. I have had experience of three London hospitals; during my residence at St. Thomas's I was much impressed by the enormous distance some of the patients came from. In the medical cases they were at all events seen by the visiting physician, but in the surgical cases they were not unfrequently operated on by the house surgeon. In the General Lying-in Hospital, Lambeth, it was the exception to admit a single woman, yet the unmarried daughter of a pharmaceutical chemist travelled from a place, the return fare to which was 14s., in order to be confined at that hospital. In addition she took lodgings in the neighbourhood until she was actually in labour. At the time I thought of the many doctors doing midwifery at £1 1s., and doing it conscientiously.

Naturally the question is asked, But why does all this abuse take place? The answer is, It is the duty of the administration to provide beds; it is the duty of the R.M.O. to fill them.

If the Houses of Parliament and Lambeth Palace were turned into hospitals I undertake to say they would very soon be filled, but where would the patients come from? No matter that they come from Ireland, Scotland, and Wales, that is no concern of the R.M.O. We hear a great deal about the overcrowding of the medical profession; but if our interests were as well protected as those of the legal profession are there would be plenty of scope for all of us.

T. W. S. Paterson, M.A., M.B.Cantab.
42 Holland Park, London, W.

